# A field investigation of the relationship between rotating shifts, sleep, mental health and physical activity of Australian paramedics

**DOI:** 10.1038/s41598-020-79093-5

**Published:** 2021-01-13

**Authors:** Wahaj Anwar A. Khan, Melinda L. Jackson, Gerard A. Kennedy, Russell Conduit

**Affiliations:** 1 Occupational Health Department, Faculty of Public Health and Health Informatics, Umm Al-Qura University, Makkah, Saudi Arabia; 2 Turner Institute for Brain and Mental Health, School of Psychological Sciences, Monash University, Melbourne, Australia; 3 Institute for Breathing and Sleep, Austin Health, Melbourne, Australia; 4 School of Health and Life Sciences, Federation University, Ballarat, Australia; 5 Psychology Discipline, School of Health and Biomedical Sciences, College of Science, Engineering and Health, RMIT University, PO Box 71, Bundoora, VIC 3083 Australia

**Keywords:** Human behaviour, Occupational health, Health occupations

## Abstract

Paramedics working on a rotating shift are at an increased risk of developing chronic health issues due to continuous circadian rhythm disruption. The acute effects of shift rotation and objectively measured sleep have rarely been reported in paramedics. This study investigated the relationships between a rotating shift schedule and sleep (using actigraphy), subjective reports of sleepiness, mood, stress and fatigue. Galvanic Skin Response, energy expenditure and physical activity (BodyMedia SenseWear Armband) were also recorded across the shift schedule. Paramedics were monitored for a period of eight consecutive days across pre-shift, day shift, night shift, and 2 days off. Fifteen paramedics (*M* age = 39.5 and *SD* = 10.7 years) who worked rotational shifts experienced sleep restriction during night shift compared to pre-shift, day shift and days off (*p* < 0.001). Night shift was also associated with higher levels of stress (*p* < 0.05), fatigue (*p* < 0.05), and sleepiness (*p* < 0.05). One day off was related to a return to pre-shift functioning. Such shift-related issues have a compounding negative impact on an already stressful occupation with high rates of physical and mental health issues. Therefore, there is an urgent need to investigate methods to reduce rotating shift burden on the health of paramedics. This could be through further research aimed at providing recommendations for shift work schedules with sufficient periods for sleep and recovery from stress.

## Introduction

There is an increasing demand for 24-h emergency medical support, which requires paramedics to work around the clock often in rotating shifts^1,2^. The adverse outcomes of night shift work have previously been reported, and there is a growing evidence indicating that shift work is becoming a serious public health issue^3^. Thus, there is an increasing need to examine the impact of shift schedules on health and well-being.

A rotating shift is a combination of a night shift and a day shift in one shift schedule, with recovery days (day off) factored in-between schedules to allow readjustment to the new roster^3^. Rotating shift work is typically adopted to reduce the amount of night work as possible, as it may impact the well-being of the employees^4^. Although there are a few advantages of rotating shifts over constant night shift, including more stable circadian rhythms and longer sleep times^5^, it has been shown to impact on a range of health and safety outcomes, including an increased risk of fatigue, accidents, stress, depression, and chronic ailments compared to day shift workers^6–10^. For example, rotating shift workers reported significantly higher insomnia and excessive daytime sleepiness compared to standard day workers^11^. Furthermore, nurses working rotating rosters reported more stress as compared to nurses worked in fixed rosters^12^, and engineers working the same shift reported poor sleep quality and significant levels of fatigue^13^. Another study reported a strong relationship between a rotating roster and fatigue and sleep problems^14^. In addition, shift rotation has also been strongly linked to poorer mental health, particularly depression and anxiety^15^. The majority of these reported the possible consequences of a rotating shift in cross-sectional studies from a chronic point of view. Little is known about the immediate or acute effects of a rotating shift on workers, especially those working in the emergency medical fields such as nurses and paramedics.

The day-to-day interaction between shift rotation and workers health has been described in a few studies that used objective measures of sleep and activity. One study from Japan reported daily sleep patterns and physical activity across an entire rotating schedule^16^. Physical activity was significantly higher during day shifts than during night shifts, and total sleep time was shorter on day shifts compared to night shifts (5.8 vs 6.4 h)^16^. Another study investigating nurses working fixed shifts indicated that the nurses’ sleep, measured using a wrist actigraphy, did not differ significantly between day and night shifts^17^. In the same study, the levels of subjective fatigue and sleepiness also did not differ significantly between day and night shifts^17^. Another study of nurses working rotating shifts reported that nurses experienced a shorter duration of sleep on both day and night shifts equally^18^. However, nurses in another study slept less and reported poorer quality of sleep during night shift compared to day shift during a rotating shift schedule^19^. Thus, rotating shift work may have a more detrimental effect on sleep than fixed shifts. There are some variations in the previous reports due to diversity in occupations, workloads, and shift types. More studies are needed to investigate the effects of rotating shift schedules on health, especially with regard to acute effects contributing to long term outcomes. In particular, paramedics, due to the traumatic and stressful nature of their work^20^, are a group that generally need more investigation.

The present study aimed to investigate the relationships between a rotating shift schedule, sleep, mood, stress, fatigue, sleepiness, energy expenditure, and physical activity levels among Australian paramedics. Paramedics were monitored for a period of eight consecutive days across pre-shift day, night shift, day shift, and two days off. It was hypothesized that during night shift day, compared to pre-shift, day shift, and days off, paramedics would report lower sleep duration, physical activity, energy expenditure, poorer mood, and higher stress, fatigue, and sleepiness.

## Method

All participants provided written informed consent prior to the study and all experimental protocols were carried out in accordance with the Declaration of Helsinki and the Australian National Statement on ethical conduct. The study was approved by the Royal Melbourne Institute of Technology Human Research Ethics Committee (ID# 21420).

## Participants

Paramedics working in Victoria, Australia were invited to participate in this study through Ambulance Employees Australia Victoria (AEAV). Participation was voluntary and limited to active full-time paramedics aged at least 18 years old, working rotating shifts and without any self-reported history of/or treatment for the following conditions: insomnia, depression, and anxiety. A single rotating shift roster consisted of two standard days shift, followed by 2 days of night shift, and then 4 days off. Before consent, paramedics were asked if they have any history of/or received treatment for insomnia, depression or anxiety via phone calls or emails, then invited to the study if the previous inclusion criteria were obtained, otherwise they were excluded from the study. All participants reported working the same rotating shift type. A power analysis was conducted based on a previous field study in healthcare workers^59^, comparing baseline nocturnal total sleep time compared to daytime total sleep time following nightshift measured with actigraphy (effect size = 0.6). It was estimated that a sample size of 23 was required to detect an effect size of 0.6, with an α = 0.05 and β = 0.8^59^.

## Materials

### BodyMedia SenseWear Armband

The BodyMedia SenseWear Armband (BSA; Jawbone, San Francisco, CA, USA) is a portable device that is connected to sensors where it can measures galvanic skin response, physical activity via steps count, and energy expenditure^21^. Galvanic skin response (GSR) is a valid mechanism to detect stress levels through changes in the electrical resistance of the skin^22^. The BSA was required to be worn on the non-dominant hand throughout the study.

### Wrist actigraphy

The Actiwatch-2 (Philips Respironics, Murrysville, PA, USA) was used for wrist actigraphy, which worn on the non-dominant wrist. The data from the Actiwatch-2 were extracted via PC using Philips Respironics software. The outcome measures used were total sleep time, sleep efficiency, number of awakenings, wake after sleep onset (WASO), bedtime, get-up time, length of time in bed, and sleep onset latency^23,24^. The outcome data from the actigraphy were scored using the sleep diary.

### Sleepiness and stress

The Karolinska Sleepiness Scale (KSS) was used to assess subjective state sleepiness on a nine-point rating scale, with higher scores indicating greater degree of sleepiness^25^. Stress was assessed using the question, ‘How stressed do you feel right now?’ answered on a five-point scale from 1 to 5 with higher numbers representing higher levels of stress.

### Positive Affect and Negative Affect Scale

The Positive Affect and Negative Affect Scale (PANAS) is a tool with 20 items developed to investigate daily positive and negative emotions^26^. The present study used an abbreviated eight-item version that had been validated in a previous naturalistic study^27^.

### Fatigue

Subjective fatigue was investigated using the Samn–Perelli Fatigue Checklist^28^. The checklist uses a ratings scale of 1–7, with higher scores indicating greater fatigue.

### The Pittsburgh Sleep Diary

The Pittsburgh Sleep Diary (PSD) is a validated self-report diary consisting of two main parts (bedtime and waketime) and 21 sub items in total that were developed to subjectively measure daily sleep quality. Daily events other than sleep were at night in the bedtime part of the diary and the wake time part to be completed just after waking from sleep^29^.

### Work related questionnaires

In addition to the subjective measurements of sleep and mood, a number of questions were asked during the day including “What was your current shift type: night/day/afternoon/on call/rest day?” “Start and end times for shift/break time and duration”, “How many hours did you spend driving for work purposes?” and “Have you encountered any significant events during the day?”.

### Procedure

**Figure 1:**
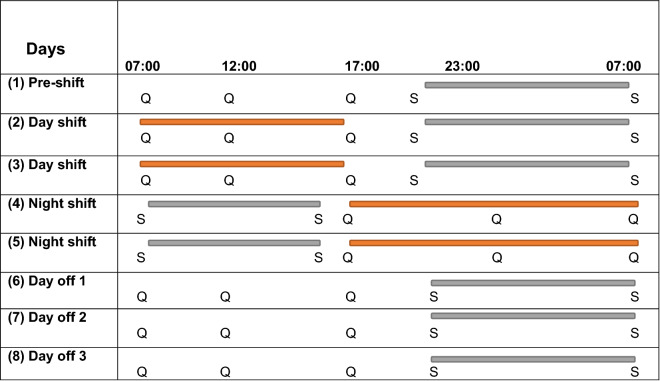
The study procedure and duration throughout a rotating shift schedule. S (Pittsburgh Sleep Diary), Q (Work diary; Samn–Perelli Fatigue Checklist, Karolinska Sleepiness Scale, and self-reported stress rating), orange bars (scheduled work time), and grey bars (sleep opportunity).

Participants were invited to take part in this study after completing a previous anonymous survey^30^. Fifteen paramedics initially accepted to participate in the study out of the 276 invitations. However three participants were excluded due to missing data of either actigraphy or diaries, leaving 12 participants (response rate 4.3%). After obtaining informed consent, participants received the BSA, actigraphy, and sleep and work diaries via post with a prepaid return envelope for returning the devices and diaries at the end of the study. Paramedics were asked to wear the BSA and the actigraphy on their non-dominant arm for a period of eight consecutive days, starting on the day before their new shift cycle to objectively record their stress and sleep (Fig. 1). During that time, participants were asked to complete the sleep diary twice a day (before sleep and after waking) and a customized work diary three times a day (before, during, and after work, or equivalent time on days off). The study investigated paramedics across five time points within their rotating shift schedules including pre-shift day, standard day shifts, night shift/s, days off one and two. Data collection started 24 h before the beginning of the shift cycle and finished 3 days after the shift ended.

### Statistical analyses

The data were analyzed using the Statistical Package for the Social Sciences (SPSS) version 24. Data distributions were adequate in terms of normality, linearity, and homoscedasticity. The data were also tested for outliers, missing values, and data entry errors. Data cleaning led to the elimination of three participants due to missing data of either actigraphy or diaries, leaving a final sample of 12 participants. Also, day shift 2, night shift 2 and day off 3 were excluded from the analysis as only 5 of the 12 remaining participants completed these days. One-way repeated measures ANOVA were used to determine the effects of shift period schedule (pre-shift; day shift 1; night shift 1; days off 1 and 2) on sleep, physical activity, GSR, energy expenditure, sleepiness, fatigue, mood, and stress. Post hoc testing was done using the Bonferroni correction. All study outcomes were measured during an entire 24-h period for each time point (e.g., total sleep time during night shift equals sleep during the entire 24-h (from 07:00 to 07:00 h) including any napping opportunity during work or outside work).

## Results

A total of 12 paramedics with complete data were included in the final data set. All paramedics were working on a rotating shift schedule. The mean age was 39.5 years (*SD* = 10.7 years), and there was a total of five men and seven women. The mean BMI was 24.5 (*SD* = 3.4).

### Sleep and sleepiness

The average total sleep time (TST; hours) that was recorded from the current sample during the entire 24-h for each day was as follows: pre-shift (*M* = 6.6, *SD* = 1.4), day shift 1 (*M* = 7.2, *SD* = 1.1), day shift 2 (*M* = 6.4, *SD* = 2.2), night shift 1 (*M* = 3.8, *SD* = 1.5), night shift 2 (*M* = 4.3, *SD* = 2.0), day off 1 (*M* = 7.2, *SD* = 1.1), day off 2 (*M* = 7.4, *SD* = 2.2) and day off 3 (*M* = 7.1, *SD* = 1.4). During night shift 1 (Day 4), five out of the 12 paramedics napped an average of 2.5 h before the beginning of night duty (daytime sleep) and the rest of sleep time was recorded during the night shift itself. The remaining participants (n = 7) slept during the night shift 1. During night shift 2 (Day 5), only one out of five slept during the day for only 1 h, while the rest slept on shift.

**Figure 2:**
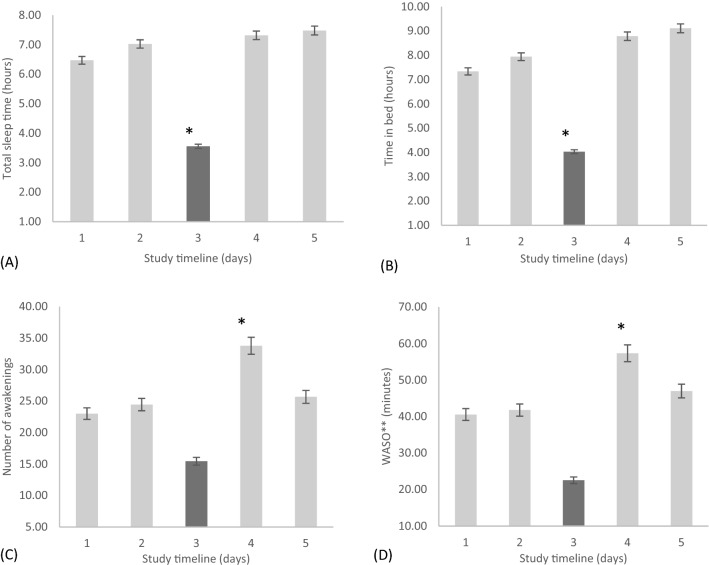
The (**A**) total sleep time in hours (daytime naps included), (**B**) time in bed in hours, (**C**) number of awakenings, and (**D**) WASO measured by actigraphy during a rotating shift schedule across 5-time points within the schedule starting from (1) pre-shift, (2) day shift one, (3) night shift one, (4) day off one, and (5) day off two. Note, *(**A**) night shift one significantly lower than all days (*p* < 0.001), (**B**) night shift one significantly lower than all days (*p* < 0.05), (**C**) day off one significantly higher than night shift one (*p* < 0.05), and (**D**) day off one significantly higher than night shift one (*p* < 0.05). **(WASO) wake after sleep onset.

A repeated measures ANOVA determined that mean TST differed significantly across the five time points in the rotating shift schedule, [*F*(2.06, 22.29) = 12.37, *p* < 0.001; η^2^ = 0.51]. Post hoc tests using the Bonferroni correction revealed that there was significantly less TST during the night shift 1 compared to pre-shift (*M* = 3.8, *SD* = 1.5 h vs. *M* = 6.6, *SD* = 1.4 h, *p* < 0.001). During the night shift 1, TST was also significantly lower in comparison to day shift 1 (*p* < 0.05), day off 1 (*p* < 0.05) and day off 2 (*p* < 0.05) (Fig. 2A).

Similarly, the mean time in bed (TIB) differed significantly across the five time points in the rotating shift schedule [*F*(2.00, 16.01) = 10.18, *p* < 0.05; η^2^ = 0.50]. Post hoc tests revealed that there was a significant reduction in TIB during the night shift 1 compared to pre-shift (*M* = 4.1, *SD* = 0.5 h vs. *M* = 7.3, *SD* = 0.5 h, *p* < 0.001). In addition, during the night shift 1, the TIB was significantly less than the day shift 1 (*p* < 0.05), day off 1 (*p* < 0.05) and day off 2 (*p* < 0.05) (Fig. 2B).

The mean number of times awakened during sleep differed significantly among the five time points in the rotating shift schedule [*F*(2.52, 20.14) = 4.736, *p* < 0.05 η^2^ = 0.278]. Post hoc tests revealed that the number of awakenings was significantly higher on day off 1 (*M* = 33.8, *SD* = 3.1; *p* < 0.05) as compared to the night shift 1 (*M* = 15.4, *SD* = 2.0) (Fig. 2C).

WASO differed significantly among the five time points in the rotating shift schedule [*F*(2.732, 21.85) = 3.93, *p* < 0.05; η^2^ = 0.23]. Post hoc tests revealed that WASO was significantly higher on day off 1 (*M* = 57.3, *SD* = 7.1 min; *p* < 0.05) in comparison to night shift 1 (*M* = 22.6, *SD* = 3.2 min) (Fig. 2D). There were no significant differences observed between the shift time points for the sleep efficiency and onset latency variables.

**Figure 3:**
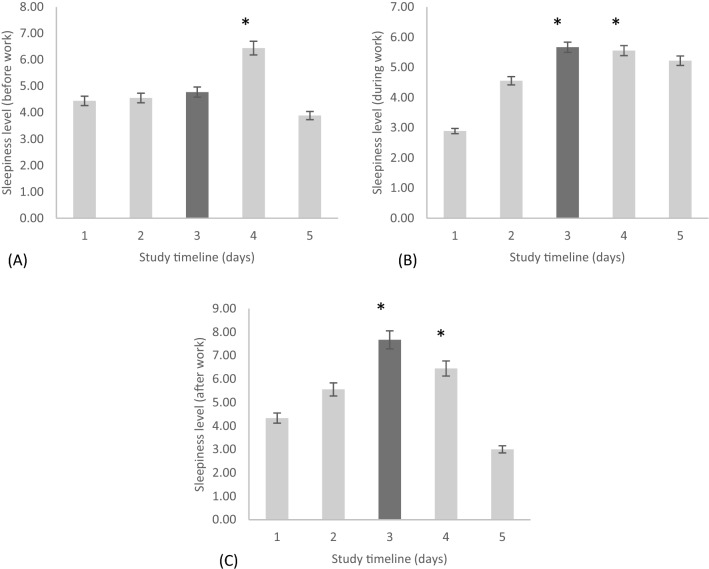
The sleepiness level reported (**A**) before work, (**B**) during work, and (**C**) after work during a rotating shift schedule across 5-time points within the schedule starting from (1) pre-shift, (2) day shift one, (3) night shift one, (4) day off one, and (5) day off two. Note, *(**A**) day off one significantly higher than day off two (*p* < 0.05), (**B**) night shift one and day off one significantly higher than pre shift (*p* < 0.05), and (**C**) night shift one and day off one significantly higher than pre-shift and day off two (*p* < 0.05).

The sleepiness score (before-work levels during work days or morning levels during non-work days) was significantly different across the rotating shift schedule [*F*(2.611, 20.887) = 4.10, *p* < 0.05; η^2^ = 0.25]. Post hoc tests showed significantly higher scores of sleepiness for day off 1 compared to day off 2 (*p* < 0.05) (Fig. 3A).

The sleepiness score (during-work levels on work days or afternoon levels during non-work days) differed significantly across the rotating shift schedule [*F*(2.70, 21.61) = 5.28, *p* < 0.05; η^2^ = 0.33]. Post hoc tests showed significantly higher scores of sleepiness on night shift 1 and day off 1 compared to pre-shift (*p* < 0.05) (Fig. 3B).

The sleepiness score (after-work levels during work days or evening levels during non-work days) was significantly different across the rotating shift schedule [*F*(2.88, 23.11) = 14.34, *p* < 0.001; η^2^ = 0.54]. Post hoc tests showed significantly higher scores of sleepiness during the night shift 1 as compared to pre-shift (*p* < 0.05). Also, sleepiness scores were significantly higher during the night shift 1 and day off 1 compared to day off 2 (*p* < 0.05) (Fig. 3C).

### Mood, stress and fatigue

The average positive and negative affect scores on the abbreviated 8-item PANAS were recorded from the current sample twice during the entire 24-h for each day. The results were as follows (before and after work): pre-shift [positive *M* = 8.2 (1.2) and *M* = 9.7 (1.6), negative *M* = 5.2 (0.5) and *M* = 6.4 (1.8)], day shift 1 [positive *M* = 10.7 (1.4) and *M* = 9.4 (1.2), negative *M* = 4.9 (0.4) and *M* = 5.4 (0.3)], night shift 1 [positive *M* = 11.0 (1.5) and *M* = 7.7 (1.4), negative *M* = 4.7 (0.4) and *M* = 5.1 (0.5)], day off 1 [positive *M* = 9.0 (1.6) and *M* = 8.4 (1.2), negative *M* = 6.0 (0.7) and *M* = 6.9 (1.5)], and day off 2 [positive *M* = 9.8 (1.3) and *M* = 7.6 (1.5), negative *M* = 5.4 (0.6) and *M* = 5.9 (0.7)]. A repeated measures ANOVA determined that mean positive and negative affect scores on the PANAS did not differed significantly across the five time points in the rotating shift schedule: positive affect (before work): [*F*(2.24, 15.70) = 1.43, *p* = 0.26; η^2^ = 0.07], positive affect (after work): [*F*(3.05, 21.35) = 1.25, *p* = 0.32; η^2^ = 0.05], negative affect (before work): [*F*(2.50, 17.54) = 1.27, *p* = 0.31; η^2^ = 0.08], and negative affect (after work): [*F*(1.32, 9.30) = 0.57, *p* = 0.51; η^2^ = 0.04].

**Figure 4:**
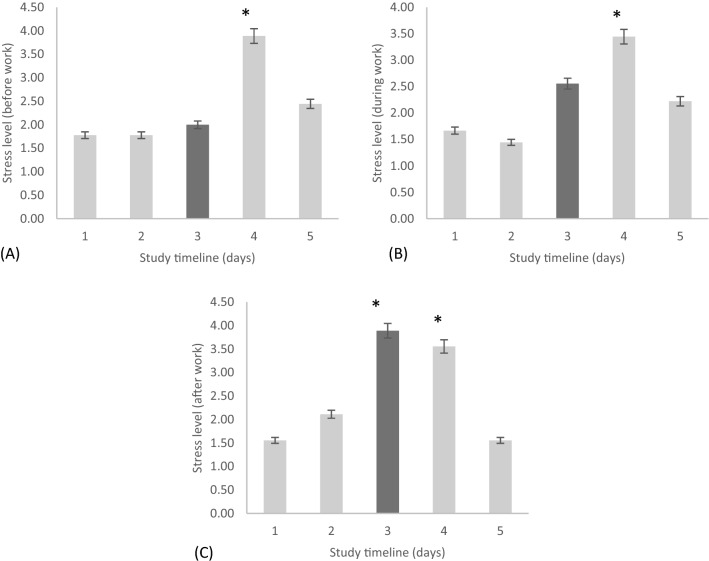
The stress level reported (**A**) before work, (**B**) during work, and (**C**) after work during a rotating shift schedule across 5-time points within the schedule starting from (1) pre-shift, (2) day shift one, (3) night shift one, (4) day off one, and (5) day off two. Note, *(**A**) day off one significantly higher than pre-shift and day shift one (*p* < 0.05), (**B**) day off one significantly higher than pre-shift and day shift one (*p* < 0.05), and (**C**) night shift one and day off one significantly higher than all other days (*p* < 0.001).

Stress (before-work levels during work days or morning levels during non-work days) differed significantly among the rotating shift schedule [*F*(2.78, 22.21) = 8.21, *p* < 0.05; η^2^ = 0.45]. Post hoc tests revealed significantly higher levels of stress on day off 1 compared to pre-shift (*p* < 0.05) and day shift 1 (*p* < 0.05) (Fig. 4A).

Likewise, stress (during-work levels on work days or afternoon levels during non-work days) differed significantly across the rotating shift schedule [*F*(2.92, 23.35) = 8.43, *p* < 0.05; η^2^ = 0.44]. Post hoc tests revealed significantly higher levels of stress on day off 1 as compared to pre-shift (*p* < 0.05) and day shift 1 (*p* < 0.05) (Fig. 4B).

Stress (after-work levels during work days or evening levels during non-work days) differed significantly across the rotating shift schedule [*F*(2.19, 17.54) = 16.85, *p* < 0.001; η^2^ = 0.63]. Post hoc tests revealed significantly higher levels of stress on the night shift 1 as compared to pre-shift (*p* < 0.05) and day off 2 (*p* < 0.05). Also, the stress level for day off one was significantly higher than pre-shift (*p* < 0.05), day shift 1 (*p* < 0.05), and day off 2 (*p* < 0.05) (Fig. 4C).

**Figure 5:**
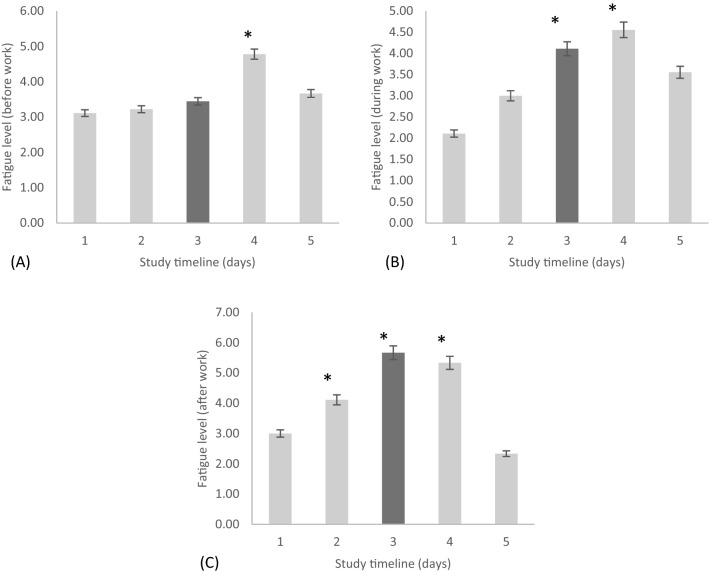
The fatigue level reported (**A**) before work, (**B**) during work, and (**C**) after work during a rotating shift schedule across 5-time points within the schedule starting from (1) pre-shift, (2) day shift one, (3) night shift one, (4) day off one, and (5) day off two. Note, *(**A**) day off one significantly higher than pre-shift (*p* < 0.05), (**B**) night shift one and day off one significantly higher than pre-shift (*p* < 0.05), and (**C**) day shift one, night shift one and day off one significantly higher than pre-shift and day off two (*p* < 0.05).

The fatigue score (before-work levels during work days or morning levels during non-work days) was not significantly different across the rotating shift schedule (*p* = 0.06; η^2^ = 0.20). However, post hoc tests showed significantly higher scores of fatigue for day off 1 in comparison to pre-shift (*p* < 0.05) (Fig. 5A).

The fatigue score (during-work levels on work days or afternoon levels during non-work days) was significantly different across the rotating shift schedule [*F*(3.10, 24.78) = 8.50, *p* < 0.001; η^2^ = 0.38]. Post hoc tests showed significantly higher scores of fatigue during the night shift 1 and day off 1 as compared to pre-shift (all *p* < 0.05) (*M* = 2.11, *SD* = 0.26) (Fig. 5B).

Fatigue levels (after-work levels during work days or evening levels during non-work days) also differed significantly across the rotating shift schedule [*F*(3.18, 25.450) = 20.450, *p* < 0.001; η^2^ = 0.66]. Post hoc tests revealed significantly higher scores of fatigue on the night shift one compared to pre-shift (*p* < 0.05), day shift one (*p* < 0.05), and day off 2 (*p* < 0.05). In addition, fatigue levels were significantly higher on day off 1 compared to pre-shift (*p* < 0.05) and day off 2 (*p* < 0.05). Also, fatigue levels were significantly higher on the day shift 1 compared to the same time on day off 2 (*p* < 0.05) (Fig. 5C).

### Physical activity

Data from the SenseWear are displayed in Table 1. The mean physical activity, measured by step count, differed significantly among the five time points in the rotating shift schedule [*F*(2.59, 23.26) = 1.40, *p* < 0.05; η^2^ = 0.37]. Post hoc tests showed that physical activity was significantly greater (*p* < 0.05) on the night shift 1 when compared to pre-shift. However, the average GSR and total energy expenditure were not statistically different across the time points in the shift schedule.

**Table 1:** Shows the means and standard errors of the data from the BodyMedia SenseWear Armband.

Variable	Pre-shift *M*(*SE*)	Day1 *M*(*SE*)	Night1 *M*(*SE*)	Day off 1 M(*SE*)	Day off 2 M(*SE*)
Average GSR (μS)	0.14 (0.02)	0.12 (0.02)	0.15 (0.04)	0.11 (0.01)	0.12 (0.02)
Steps	6049 (1219)	6548 (1009)	9061 (1125)*	7777 (1468)	6639 (1765)
Total energy expenditure (kJ)	8292 (1137)	9386 (798)	11,836 (664)	10,389 (1322)	9480 (1226)

GSR (Galvanic skin response), μS (microsiemens), and kJ (kilojoules). *Night shift was significantly higher compared to pre-shift (*p* < 0.05).

## Discussion

This field study investigated paramedics across an entire rotating shift schedule, starting with the pre-shift day, day shift, night shift, and 2 days off. The study investigated sleep (actigraphy and PSD), physical activity (BSA), total energy expenditure (BSA), mood (PANAS), sleepiness (KSS), fatigue (Samn–Perelli Fatigue Checklist), and stress (BSA and a self-reported stress scale). The actigraphy data revealed significantly less TST during the night shift one when compared to the pre-shift day, day shift one, and days off. The number of awakenings and WASO were significantly higher during day off one compared to the night shift. Sleep efficiency and onset sleep latency were not significantly different across the rotating shift schedule. However, paramedics reported significantly higher levels of sleepiness and fatigue during and after the night shift, and across day off one, as compared to pre-shift and the second day off. Levels of stress yielded a similar trend, as paramedics reported significantly higher stress during and after the night shift, and across day off one, when compared to pre-shift, day shift one, and day off two. Significantly more physical activity, measured by step count, was observed during the night shift as compared to pre-shift. Total energy expenditure, GSR (stress) and mood did not change significantly during the rotating shift schedule.

During the first night shift, the paramedics recorded an average of 3.8 h of TST, which was significantly less than the pre-shift day, day shift, and days off. It is plausible that since many of these paramedics were only working one night shift and were transitioning to off days following this shift, they did not attempt to obtain a full 7–8 h of sleep during this period. Five of the paramedics were rostered to a second night shift (Day 5), and recorded slightly more sleep after the night shift, with an average TST of 4.3 h, which is still considerably lower than reported in other shift worker populations. For example, nurses in the United States^17^ and Japan^31^ slept an average of 5.2 h after working a night or evening shift. However, airline ground crew slept an average of 4.3 h after the night shift, which is similar to what was recorded from our study^32^. Even under ideal conditions, simulated night shift studies have shown that sleep after a night shift is severely truncated, which is likely due to the impact of circadian processes in the early afternoon disrupting sleep^33^. The recorded TST in our sample of paramedics reflects a period of severe sleep restriction after night shift day that was also shorter than the TST measured in other shift worker populations^17,31,32^. While it should be noted that this was only in a small number of paramedics in our sample, it does raise concerns given the known impact of sleep restriction on decision making and cognitive functioning more generally^34^.

Other sleep parameters measured by the actigraphy were WASO and the number of awakenings, which were both significantly higher during the first day off compared to night duty. Although not significant, but still considerably high, WASO and the number of awakenings were recorded during the second day off compared to pre-shift measurements. In a previous study using actigraphy, a higher WASO was found a day after working an afternoon shift among midwives^35^. Also, nurses working evening shifts scored higher WASO compared to non-shift workers^31^. A greater number of awakenings were reported by rotating shift workers, especially following a shift change, in comparison to workers with fixed shifts^36^. It seems that paramedics and shift workers from different cohorts share a common sleep pattern where a high WASO and number of awakenings were recorded primarily after night duty or a shift change. Higher WASO and number of awakenings indicates sleep fragmentation which can be an indicator of elevated stress^37,38^. It is also likely to be the result of unstable and disrupted circadian rhythms, from rapidly switching between day and night schedules. This is one of the adverse effects of working rotating shifts and switching from night to day sleep and back to night sleep every week.

The levels of stress, fatigue, and sleepiness were significantly higher during days off as compared to pre-shift measurements. It is plausible that the restricted sleep during night duty (3.8–4.3 h) contributed to the increases in stress, fatigue, and sleepiness during this time. Previous studies have linked sleep restriction with stress in physicians and nurses^39,40^. In fact, a study that restricted sleep in a laboratory found that a shorter duration of sleep was strongly related to higher levels of stress^41^. Also, fatigue and sleepiness were strongly linked to shorter TSTs in nurses, physicians, and airline ground crew^17,31,32,42,43^. Although the signs of stress, fatigue, and sleepiness were not significantly higher during the second day off when compared to other time points, the levels were still considerably high especially in the beginning and in the middle of day off two compared to pre-shift. In nurses, the signs of stress and fatigue continued beyond night duty, as they reported significant signs of fatigue and stress during 2 days off work after night duty^44^. Also, airline cabin crew reported significant signs of sleepiness for 2 days after the end of the shift^45^. The findings of the current study are consistent with previous findings, where subjective levels of stress, fatigue, and sleepiness are impacted by a shorter sleep duration. The effect continues to impact workers during off days, and the levels were still considerably high 1–2 days after the end of the shift.

The level of physical activity, which was measured by step count, was significantly higher during night duty compared to pre-shift. However, rotating shift workers from Japan reported significantly higher physical activity during day compared to night shifts^16^. Another recent report compared day and night shift healthcare workers indicated no differences in physical activity as measured for 24-h period for both groups^46^. While more steps count or physical activity typically represent a healthy lifestyle, in the present study, this finding is probably due to the extended awake time and less rest/nap opportunities for paramedics during night duty. It may lead to fatigue and exhaustion, thus impacting their performance or safety^47^. Despite the fact that more physical activity is linked to better well-being and mental health as well as less fatigue in the general population^48^, engaging in more physical activity while working against the normal body clock may lead to physical exhaustion^49^.

The mood of the participants was measured by the PANAS and did not show significant changes across the shift schedule. This finding suggests that a rotating shift has no relationship with the paramedics’ mood. The mood issues reported by previous studies in paramedics may be due to the cumulative effect of shift work^2,30,50,51^. Based on the findings from the current sample of paramedics, a rotating shift may be related to sleep duration, stress, fatigue, and sleepiness levels but not mood.

In addition to the relationship with stress and fatigue, lack of sleep has been associated with a decline in cognitive function, judgment, and decision-making^52^. In fact, less than 4 h of TST during night shift simulated in a laboratory was linked to significant deficits in cognitive function when compared to groups that slept more than 5 h, suggesting a dose–response relationship between sleep duration and performance^53^. Certainly, cognitive functions and performance are two of the most important abilities for healthcare shift workers, especially paramedics. Any deficits in those abilities can compromise the safety of both the workers and their patients. Future studies should focus on assessing paramedics’ performance and cognition during shift work and on off days as well.

Recovery from shift work is crucial in maintaining worker’s health^54^. The current rotating shift schedule for paramedics in Victoria, Australia includes 2 days of standard day shifts followed by 2 days of night shifts and then 4 days off work or recovery in-between every other roster. The amount of recovery needed in-between shifts may differ from one group of workers to another, as nurses from a recent report required at least 3 days of recovery^44^. Non-shift workers usually require no longer than 2 days to recover^55^. Also, the duration of work is an important factor in determining the amount of recovery^56^, as longer working hours are attributed to the need for longer recovery times^57^. Outcomes of the present study seem to show improvement from day off 2, although not to pre-shift levels. The current study did not assess these outcomes beyond day off 2, therefore we do not know if 4 days of recovery is sufficient for sleep, mood and stress to return to pre-shift levels in this population. Future studies should focus on days where shift workers required to change their sleep habits, starting from night shifts until the second or third day of recovery after night work, as these appear to be the most challenging days for them.

Fixed shifts would be a solution that avoids the problems of continually having to adjust between different shifts. This could be done on the basis of assigning people to shifts based on their chronotype^58^. Chronotype was assessed in 136 ambulance workers and found that while it would be possible to staff the day shift, there would always be a shortage of staff that are true evening type to staff the night shift (evening types = 11%)^30^. It may be difficult to fill workers into a fixed night shift, but it would be advantageous because they only have to adapt to a fixed work schedule and any difficulties in adjusting would result from social demands that may conflict with their permanent shift schedules^59^. Fixed night shift is detrimental to health and fixed day shift is not possible in some essential services that require 24 h coverage e.g. police, paramedics. Thus, rotating shifts have been adopted.

The present study assessed paramedics through their entire rotating roster and off days using wrist actigraphy to record their sleep and objective information regarding physical activity and subjective levels of stress, fatigue, and sleepiness. Sample size was not large enough to conduct further analyses to investigate the outcomes of the study during the day shift 2, night shift 2 and day off 3. However, from the observed TST during night shift 2, we can speculate similar outcomes to what found during night shift 1. Although our sample size was small, this was due to the intensive assessment of day-to-day data in the field. Further studies in larger samples of paramedics are required to confirm the current findings.

Being a field study, the shift comparisons were restricted by the shifts that were running, with night shifts being 4 h longer than day shift. Ideally, a comparison of shifts of equal duration would be best from an experimental perspective, however it could be argued that these results have ecological validity, reflecting outcomes from shift schedules used in the industry.

This study did not assess employment history, which may be a relevant factor related to these findings, as newly recruited paramedics may react differently than paramedics with experience to rotating shift conditions. The subjective stress rating used was a single question assessing the current state of stress, and it has not been previously validated. In addition, the study did not investigate whether the pre-shift day represented day off 4 from a previous shift or another day (e.g., last day of a vacation).

Although the current study was sufficiently powered to reveal statistically significant findings, with moderate to high effect sizes, the sample size was small (n = 12). Additionally, within this small sample there was a considerable amount of missing data. Thus, the self-reported measures used in the study may be particularly prone to a response bias. However, this data was sampled from first-response paramedics in a highly stressful and demanding work environment, where lives can be at risk. Therefore, we believe such study conditions are highly susceptible to missing data.

In conclusion, this study investigated paramedics across a single rotation of their shift schedule, including two off days. The duration of sleep measured using actigraphy was significantly less during night duty when compared to pre-shift, day shift, and off days one and two. The levels of stress, fatigue, and sleepiness were all related to the sleep restriction that happened during night duty. These levels started to increase significantly after the end of the night duty and peaked significantly throughout day off one. The highest level of physical activity was detected during 24 h periods that included night shift. During these periods paramedics may be at risk of physical fatigue and exhaustion. Therefore, it was observed that working on a rotating shift was associated with sleep restriction, higher stress, fatigue, and sleepiness levels, which together may be detrimental to a worker’s health. Given these preliminary results, there is a need to investigate methods to reduce rotating shift burden on health with a particular focus on night shifts and days off.
